# Low Levels of Chito-Oligosaccharides Are Not Effective in Reducing Deoxynivalenol Toxicity in Swine Jejunal Explants

**DOI:** 10.3390/toxins10070276

**Published:** 2018-07-04

**Authors:** Juliana Gerez, Letícia Buck, Victor Hugo Marutani, Caroline Maria Calliari, Ana Paula Bracarense

**Affiliations:** 1 Laboratory of Animal Pathology, Universidade Estadual de Londrina, Campus Universitário, Rodovia Celso Garcia Cid, Km 380, Londrina, Paraná 86057-970, Brazil; julianarubira@hotmail.com (J.G.); leleyamasaki26@gmail.com (L.B.); vhbmarutani@gmail.com (V.H.M.); 2 Academic Department of Food, Universidade Tecnológica Federal do Paraná, Avenida dos Pioneiros, 3131, Londrina, Paraná 86036-370, Brazil; calliari@utfpr.edu.br

**Keywords:** functional oligosaccharides, mycotoxins, swine, explant technique, intestinal morphology, goblet cells

## Abstract

Deoxynivalenol (DON) is a mycotoxin that affects the intestinal morphology of animals, impairing nutrient intake and growth. On the other hand, dietary supplementation with functional oligosaccharides as chito-oligosaccharides (COS) has shown positive effects on the intestinal health of piglets. Therefore, the objective of the present study was to evaluate the effect of low doses of COS in preventing DON-induced intestinal histological changes, using a swine jejunal explant technique. The intestinal explants were incubated at 37 °C in culture medium for 4 h and exposed to the following treatments: (a) control (only culture medium), (b) DON (10 µM), (c) 25COS (0.025 mg·mL^−1^ of COS); (d) 50COS (0.05 mg·mL^−1^ of COS); (e) 25COS plus DON (25COS + DON); (f) 50COS plus DON (50COS + DON). Explants exposed to COS presented intestinal morphology similar to control samples. DON induced a significant decrease in the histological score as a consequence of moderate to severe histological changes (apical necrosis, villi atrophy, and fusion) and a significant decrease in morphometric parameters (villi height, crypt depth, villi height:crypt depth ratio, and goblet cells density). The intestinal morphology of samples exposed to COS + DON remained similar to DON treatment. In conclusion, low levels of COS did not counteract DON-induced intestinal lesions.

## 1. Introduction

Deoxynivalenol (DON) is produced mainly by *Fusarium graminearum* and *Fusarium culmorum* in cereals as wheat, barley, and maize [1]. Processing methods may reduce the amount of DON in cereals, however, this mycotoxin is not completely eliminated in grains intended for animal and human consumption [2,3]. In a survey including 15,549 samples of cereals from European and Asian countries, DON was the most prevalent mycotoxin, with concentrations ranging from 0.250 to 50.289 mg·kg^−1^ and a mean level of 0.967 mg·kg^−1^ [4]. This fusariotoxin is known to affect the functional morphology of the intestinal tract in animals, compromising the absorption of nutrients by the intestinal epithelium [5,6]. Consequently, DON can result in significant economic losses in animal production due to the adversely altered animal performance [7,8].

On the other hand, the gut health-promoting effects of chitosan oligosaccharides in swine nutrition have been broadly acknowledged [9]. Chito-oligosaccharides (COS) are obtained by depolymerization of chitin or chitosan by the action of acids, enzymes, or even physical methods [10]. Chitosan is initially extracted from the shells of crustaceans (e.g., shrimp and crabs) or from the cell walls of fungi. However, it has been suggested that COS produced through fermentation of microorganisms such as *Bacillus* spp. [11], using chitosan as a carbon source, can lead to more standardized results since this biotechnological means of obtaining it is independent of climate and environmental changes [12]. Radicals of *N*-acetylglucosamine present in the molecule of chitosan, and in its derivatives are responsible for diverse biological activities [13]. The results of many studies point to antibacterial [14] and anti-inflammatory properties [15] in COS in addition to anti-oxidative, antitumor, and immunostimulatory effects [16]. In pigs, COS modulates the gut microbiota, favoring the growth of beneficial bacteria [17,18]. In addition, diets supplemented with COS result in beneficial effects on small intestinal morphology, barrier function, nutrient digestibility, and zootechnical parameters of weanling pigs [18,19]. Due to these properties, chitosan and COS have been considered as an effective prebiotic and a potential alternative to antibiotics in pig nutrition [17,20,21,22].

Although COS-treatment has shown beneficial effects in weaners, so far, there have been few reports on the action of oligosaccharide derivatives during intestinal exposure to mycotoxins [23]. Given the need to broaden the knowledge about the possible protective effect of functional oligosaccharides on DON-induced intestinal toxicity in piglets, the objective of the present study was to evaluate the effects of different doses of COS in pig jejunal explants exposed to DON.

## 2. Results

### 2.1. Histological Evaluation of the Explants Exposed to COS and DON

**Figure 1:**
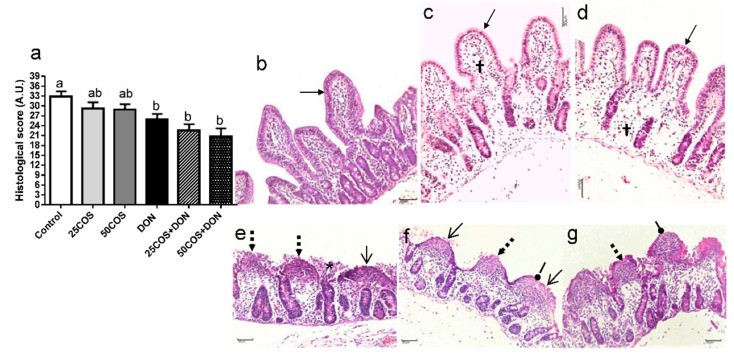
Histological evaluation of the explants exposed to chito-oligosaccharides (COS) and deoxynivalenol (DON). (**a**) Values of histological scores of swine jejunal explants exposed to control treatment (□), 0.025 mg·mL^−1^ of COS (25COS) (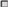), 0.05 mg·mL^−1^ of COS (50COS) (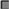), DON (10 µM) (■), 25COS plus DON (25COS + DON) (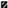), and 50COS plus DON (50COS + DON) (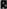). Values are mean ± SEM. Means with unlike letters (^a, b^) differ significantly by Tukey’s test (*p* ≤ 0.05). Maximum histological score of 39 points in A.U. (arbitrary units); (**b**) Explants exposed to control (n = 30); (**c**) 25COS-exposed explant (n = 30); (**d**) 50COS-exposed explant (n = 30); (**e**) DON-exposed explant (n = 30); (**f**) Explant exposed to treatment 25COS + DON (n = 30); (**g**) Explant exposed to treatment 50COS + DON (n = 30). Histological endpoints with different arrows: simple columnar epithelium (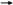), moderate edema of the lamina propria (†), multifocal to diffuse fusion and atrophy of villi (→), discontinuous epithelium (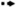), necrotic debris (*), and severely flattened epithelial cells (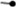) (Bar = 50 μm; Hematoxylin and eosin staining).

The treatments with 25COS and 50COS resulted in no significant change on the histological score (Figure 1a). Explants exposed to COS showed moderate edema of the lamina propria and simple columnar epithelium was preserved (Figure 1c,d). After 4 h of exposure to DON, explants presented a significant decrease of 21.22% in the histological score in relation to control samples (*p* = 0.044) (Figure 1a). Explants submitted to DON showed fusion and atrophy of villi with discontinuous epithelium exhibiting severely flattened enterocytes with necrotic debris (Figure 1e). COS did not affect DON-induced lesions, and a significant reduction in histological scores of 31.25% (*p* = 0.013) and 36.64% (*p* = 0.003) was also observed in the intestinal tissue exposed to 25COS + DON and 50COS + DON when compared with the control, respectively (Figure 1a,f,g).

Villi height was a sensitive parameter of intestinal health; a decrease around 37.29%, 41.45%, and 37.87% in this parameter was observed after exposure to DON (*p* = 0.003), 25COS + DON (*p* < 0.0001), and 50COS + DON (*p* < 0.0001) in relation to control samples, respectively. Mitotic figures were observed in crypt epithelium, and crypt depth was maintained in all experimental groups. In accordance with the above results, the villi height:crypt depth ratio was significantly reduced in DON-treated samples and COS + DON-treated explants in comparison to control explants (*p* < 0.05). The samples exposed to treatments with COS showed no significant changes on morphometrical parameters (Table 1).

### 2.2. Goblet Cell Density of the Explants Exposed to COS and DON

**Figure 2:**
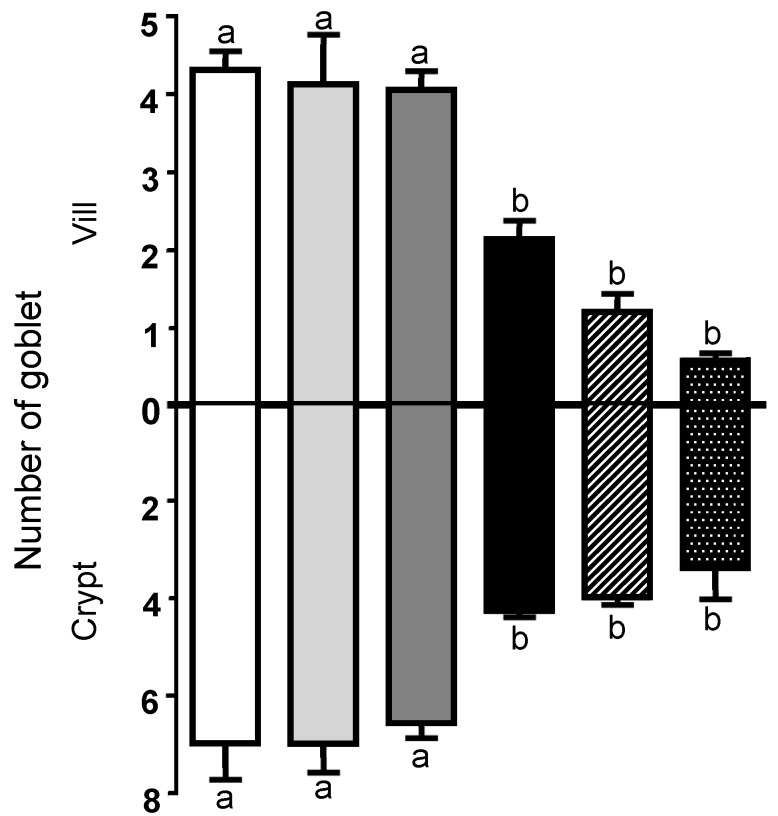
Mean goblet cell number on villi and crypts of swine jejunal tissue submitted to different treatments: control (□), 0.025 mg·mL^−1^ of COS (25COS) (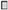), 0.05 mg·mL^−1^ of COS (50COS) (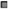), DON (10 µM) (■), 25COS plus DON (25COS + DON) (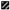), and 50COS plus DON (50COS + DON) (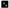). Values are mean ± SEM represented by vertical bars. Means with unlike letters (^a, b^) differ significantly by Tukey’s test (*p* < 0.05).

Goblet cells are present in the intestinal epithelium and are responsible for synthesis and secretion of mucins. Explants exposed to COS presented a number of goblet cells similar to control treatment. The goblet cell density in explants exposed to DON was significantly decreased in the villi region compared to the control samples (*p* = 0.003). The reduction was more pronounced in the samples exposed to 25COS + DON (43.46%; *p* < 0.0001) and 50COS + DON (68.57%; *p* < 0.0001) when compared with control group. In relation to goblet cell density in crypts, explants exposed to DON, 25COS + DON, and 50COS + DON showed a significant decrease of 38.97% (*p* = 0.008), 42.98% (*p* = 0.003), and 51.57% (*p* = 0.001), respectively, compared with the explants exposed to control treatment (Figure 2).

## 3. Discussion

Functional oligosaccharides such as COS have been widely suggested as dietary supplements in post-weaning diets of piglets. While COS have been shown to improve growth performance, immunological status, gut microbiota, and intestinal morphology in weaned pigs [9], their effects on mycotoxins-induced intestinal toxicity have not been yet explored. Due to global distribution of DON, its permanence through feed and food processing, and its known intestinal toxicity, DON is considered a food security concern [3,5,24]. Studies on the potential protective effect of feed additives on the mycotoxins-induced intestinal toxicity have been conducted by our research group [25]. In the present study, we have assessed whether COS can prevent DON-induced intestinal toxicity using an ex vivo approach. Intestinal explants represent an adequate alternative model to toxicological studies [26]. This technique preserves the histological structure observed in vivo and allows for the reduction in the number of experimental animals [27]. To the best of our knowledge, it is the first work to analyze the action of COS facing a fusariotoxin challenge in piglets.

In previous studies, the effects of COS on intestinal health have been evaluated in piglets [9]. The range of COS effective doses is large (30 mg·kg^−1^~5000 mg·kg^−1^), and the results are variable [11,28]. In this study, the treatment with low levels of COS for 4 h induced no effects on the intestinal morphology (histological score, villi height, crypt depth, and villi height:crypt depth ratio). In accordance with those results, we demonstrated in a previous study that increasing doses of COS (0.025, 0.05, 0.10, and 0.15 mg·mL^−1^) induced no change on the intestinal morphology of swine explants [29]. Accordingly, no effect on intestinal morphology was observed in weaned pigs fed diets containing increasing levels of COS (30 to 600 mg·kg^−1^) for 14 days [18,28]. However, in piglets fed diets supplemented with 150 and 200 mg COS·kg^−1^ of feed for a 21-d and 26-d period, respectively, a significant increase on villi height and villi:crypt ratio was observed in jejunum of animals [17,19]. Although the mechanisms involved in COS action have not yet been fully elucidated,*N*-acetyl glucosamine, a basic component of this molecule can play an important role [13]. This radical can bind to determined strains of pathogens (bacteria), interfering with their adhesion capacity to the intestinal mucosa [11,30]. Moreover, in vivo studies indicate that COS is rapidly and efficiently utilized by beneficial intestinal microorganisms, increasing the proportions of *Lactobacillus* spp. and *Bifidobacterium* spp., especially in weaning pigs [17,30]. Thereby, changes in intestinal microbiota composition may provide a favorable environment for the proliferation of enterocytes as reported recently by Suthongsa et al. [19] and Thongson et al. [31]. Thus, short periods of treatment with COS would be insufficient to stimulate the intestinal cell proliferation and improve gut morphological parameters in weaned piglets.

Regarding animal feed and human food, the intestine is the first organ to be exposed to the toxic effect of mycotoxins [32]. In this study, DON induced a significant decrease in the histological parameters (histological score, villi height, crypt depth, and villi height:crypt depth ratio). The main histological changes were atrophy and fusion of the villi, loss of apical enterocytes, necrotic debris, and severe flattening of columnar epithelium. These results agree with previous in vivo and ex vivo studies that have evaluated intestinal exposure to DON [5,25,33]. DON at the cellular level causes inhibition of protein synthesis [34], inducing oxidative stress and cell apoptosis [35].

Co-treatment of COS and DON for 4 h did not prevent the lesions caused by DON as observed in the histological score (villi height, crypt depth, and villus:crypt ratio). It is important to highlight that studies about the effects of COS on the intestinal toxicity of DON are scarce, and in the available databases, no study concerning this aspect was found. Previous studies have evaluated the adsorption capacity of chitosan and chitosan polymers on mycotoxins in in vitro models. Recently, Solís-Cruz et al. [36], using an in vitro gastrointestinal model for poultry, identified that chitosan showed a lower binding activity against DON when compared to cellulosic polymers. Additionally, a poor adsorption efficiency of chitosan polymers for DON and T-2 toxin has been observed in a buffer system [37]. No results about the adsorption capacity of COS on DON was found. Thereby, we hypothesize that COS is not effective in reducing DON toxicity in swine jejunal explants due to its poor adsorbent capacity for mycotoxins. Moreover, it is important to note that short periods of exposure to COS can be insufficient to influence the intestinal population of *Lactobacillus*, which can counteract the adverse effects of DON in weaned piglets [38].

Considering that oligosaccharides from different natural sources show similar prebiotic activities in animals [39], it is important to note that the protective effect of milk-derived oligosaccharides (galacto-oligosaccharides (GOS)) has been identified on intestinal barrier function in a DON challenge. In that work, the pretreatment with GOS was responsible for preventing the decrease of DON-induced transepithelial electrical resistance in the Caco-2 cell monolayer, while the co-incubation of GOS and DON did not reduce the DON-induced intestinal barrier disruption. Similarly, when mice received GOS before DON, villi height of the proximal small intestine remained comparable to the control animals [40]. Thus, the treatment with functional oligosaccharides before exposure to mycotoxins is an important factor to consider in future studies.

Mucins secreted by goblet cells form the mucus layer present on the gastrointestinal mucosal surface and prevent adhesion of pathogens to enterocytes [41]. COS showed no effect on the goblet cell density of intestinal explants. Similarly, COS-supplemented diets (30 or 100 mg·kg^−1^) induced no changes on goblet cell density in piglets fed for a period of 14 and 18 days, respectively [28,42]. In accordance, villi and crypts of swine intestinal explants exposed to increasing levels of COS (0.025, 0.05, 0.10, and 0.15 mg·mL^−1^) showed no difference on goblet cell density among the treatments [29]. Conversely, 300 mg/kg of chitosan for 21 days resulted in a significant increase on the goblet cell density in the jejunal villi of weaned piglets fed a supplemented diet and challenged with enterotoxigenic *Escherichia coli* during a preliminary trial period [22]. Probably, higher doses of COS are necessary to induce changes on goblet cells density. On the contrary, DON induced a significant decrease on the number of goblet cells in intestinal explants. Reduction in the goblet cell density after a challenge with DON has already been reported in pigs and cultures of swine jejunal tissue [5,33,43]. While the effect of toxic doses of DON (10 µM) on goblet cells have been related to cell death in intestinal crypts [25], subtoxic doses of DON (1 µM) induced a reduction in the mucin production by down-regulation of the gene expression of these glycoproteins [44].

In this study, COS did not prevent the reduction in the goblet cell density induced by DON. Interestingly, a more pronounced decrease was observed on the goblet cell density in the villi of explants exposed both to COS and DON in relation to the other treatments. Previous studies have shown that chitosan induces a disruption of tight junctions (TJs) (CLDN4, zonula occludens 1 and occludin) affecting the epithelial permeability in the Caco-2 cell monolayer. These changes were transient and reversible after chitosan removal [45,46]. Similarly, Xiong et al., [28] showed that the expression of occludin in ileum and zonula occludens 1 in jejunum and ileum were decreased in piglets fed a diet supplemented with 30 mg·kg^−1^ of COS for a 14d-period. Similarly, in in vivo and ex vivo studies, DON induces a significant decrease in the expression of E-cadherin and occludin in the intestine of piglets [5,33]. Accordingly, considering our results and these previous studies, we hypothesize that COS acts similarly to DON on intestinal explants by regulating TJ expression, resulting in increased epithelial permeability, cell degeneration, and death.

In conclusion, we have shown that the exposure to low levels of COS induced no reduction in the toxic action of DON on the intestinal histological structure. The use of dietary supplements in animal feed with the objective of minimizing the toxic action of mycotoxins on intestinal tissue is of increasing interest. Due to the lack of results in the literature, additional studies to assess the effect of functional oligosaccharides on DON-induced intestinal toxicity are necessary. Experimental factors such as exposure time and host microbiota should be considered in future analyses.

## 4. Materials and Methods

### 4.1. Animals

After weaning, five crossbred (Landrace × Large White × Duroc) piglets were allocated in separate bays and received a standard diet from 21 to 24 days of age. Feed and water were provided *ad libitium*. A sample of feed was analyzed for mycotoxins by high performance liquid chromatography. Aflatoxins (B1, B2, G1, and G2), fumonisins B1 and B2, zearalenone, and deoxynivalenol were below the limit of detection (data not shown). After 3 days of feeding with solid feed, the animals were submitted to euthanasia using sodium pentobarbital (40 mg·kg^−1^ of body weight (BW)) intravenously. The techniques used in the procedures were previously approved by the Institutional Ethics Committee for Animal Experimentation (number 11361.2014.30).

### 4.2. Chito-Oligosaccharides

Mild chrysalis flour of the silkworm Bombyx mori L. (0.7 g·L^−1^) was provided as the only carbon (chitosan) source and peptone (0.3 g·L^−1^) as the nitrogen source to Bacillus subtilis DP4. The fermentation occurred at pH 9.6, at 28 °C, under agitation (110 rpm) for 96 h. The bacterial biomass was separated by centrifugation, and the supernatant was sterilized to be used in the assays as a crude extract containing 6.48 mg·mL^−1^ COS [29]. COS quantification was determined by the MBTH technique [47] using *N*-acetylglucosamine (0 to 100 µM) as standard.

### 4.3. Culture of Explants and Exposure to DON and COS

The procedures for obtaining jejunal explants followed the previously described technique [25]. Briefly, jejunum segments of 5 cm were washed with phosphate buffered saline (PBS), opened longitudinally, and explants sampled using a biopsy punch of 8 mm. Three explants per well were placed in six-well plates (EasyPath, São Paulo, Brazil) containing Dulbecco’s modified Eagle’s medium (DMEM-Gibco, Gaithersburg, MD, USA), antibiotics (penicillin/streptomycin—1.25 µL·mL^−1^, Gibco and gentamicin—10 µL·mL^−1^, Novafarma), fetal bovine serum (100 µL·mL^−1^—Invitrogen, Carlsbad, CA, USA), and L-glutamine (0.4 µL·mL^−1^—Sigma-Aldrich, São Paulo, Brazil). Explants were incubated at 37 °C under orbital shaking for 4 h in the following experimental groups: (1) Control: culture media; (2) DON: culture media with 10 μM of DON (D0156, Sigma-Aldrich); (3) 25COS: culture media with 0.025 mg·mL^−1^ of COS; (4) 50COS: culture media with 0.05 mg·mL^−1^ of COS; (5) 25COS + DON: culture media with 25COS plus DON; (6) 50COS + DON: culture media with 50COS plus DON. The dose of DON used in this study was equivalent to an ingestion of feed contaminated with 3 mg DON·kg^−1^ of feed and was considered toxic in in vivo [5] and ex vivo [48] studies. The concentrations of COS used in this experiment corresponded to an ingestion of 25 mg COS·kg^−1^ and 50 mg COS·kg^−1^ of feed. Six explants (replicates) from each animal were collected for each treatment. After this period, explants were fixed in a 10% buffered formalin solution for morphological and morphometric evaluation.

### 4.4. Morphological and Morphometric Assessment

Samples for histological analysis were dehydrated in increasing concentrations of alcohol, diaphanyzed, and embedded in paraffin. Tissue slices (5 µm thickness) were stained with hematoxylin and eosin for morphological and morphometric evaluations. The periodic acid of Schiff (PAS) stain was used to assess the number of goblet cells in 10 villi and 10 crypts/explant at 400× magnification. The histological score previously described by Maidana et al. [49] and Gerez et al. [29] was used to compare histological changes between the different experimental groups. Briefly, the criteria used were enterocyte morphology, apical denudation of villi, changes in the lamina propia, villi fusion and atrophy, number of villi, and cell debris [43]. Samples with well-preserved histological structure showed a maximum score of 39 points, while the minimum score of 0 points was assigned to the explants with severe and diffuse lesions. For morphometric evaluation, villi height and crypt depth were measured using an image software system (Motic Image Plus 2.0, Motic Microscopy, Kowloon, Hong Kong) with 200× magnification as previously described [43]. The villi-height:crypt-depth ratio was also considered in the evaluation of intestinal integrity.

### 4.5. Statistical Analysis

A completely randomized design with five repetitions per treatment was employed. Six replicates from each animal were analyzed, resulting in one repetition. The data were represented as means ± SEM (standard error of the mean) and analyzed by free software Rstudio version 1.1.442—© 2009–2018 (Boston, MA, USA). Assumptions of residual normality (Shapiro-Wilk’s test) and homoscedasticity (Bartlett’s test) were checked and the data were submitted to analysis of variance (ANOVA) followed by Tukey’s test. *p*-value ≤ 0.05 was regarded as statistically significant.

## Figures and Tables

**Table 1:** Morphometrical evaluation of the swine jejunal explants exposed to COS and DON (mean ± SEM).

Treatments	Villi Height, µm	Crypt Depth, µm	Villi Height:Crypt Depth, μm:μm
Control	139.68 ± 8.48 ^a^	129.21 ± 8.53 ^a^	1.09 ± 0.07 ^a^
25COS	121.74 ± 10.50 ^ab^	136.69 ± 16.11 ^a^	0.90 ± 0.05 ^ab^
50COS	116.56 ± 13.71 ^ab^	110.77 ± 3.34 ^a^	1.04 ± 0.09 ^a^
DON	87.60 ± 4.01 ^b^	140.86 ± 18.60 ^a^	0.64 ± 0.06 ^b^
25COS + DON	81.79 ± 9.99 ^b^	131.09 ± 8.18 ^a^	0.63 ± 0.09 ^b^
50COS + DON	86.78 ± 7.50 ^b^	132.34 ± 13.39 ^a^	0.65 ± 0.02 ^b^

Means (n = 30) within a column followed by the different superscripts (^a, b^) differ significantly by Tukey’s test (*p* < 0.05). Explants exposed to Dulbecco’s modified Eagle’s medium (Control), 0.025 mg·mL^−1^ of COS (25COS), 0.05 mg·mL^−1^ of COS (50COS), DON (10 µM), 25COS plus DON (25COS + DON), or 50COS plus DON (50COS + DON).
